# Pediatricians’ focus of sight at pain assessment during a neonatal heel puncture

**DOI:** 10.1590/1984-0462/2024/42/2023089

**Published:** 2023-12-11

**Authors:** Erica Souza Silva, Marina Carvalho de Moraes Barros, Julia Baptista Lopes Borten, Lucas Pereira Carlini, Rita de Cássia Xavier Balda, Rafael Nobre Orsi, Tatiany Marcondes Heiderich, Carlos Eduardo Thomaz, Ruth Guinsburg

**Affiliations:** a Universidade Federal de São Paulo, Escola Paulista de Medicina, Departamento de Pediatria, Disciplina de Pediatria Neonatal – São Paulo, SP, Brasil.; b Centro Universitario FEI, Departamento de Engenharia Elétrica, Laboratório de Processamento de Imagens – São Bernardo do Campo, SP, Brasil.

**Keywords:** Infant, newborn, Pain measurement, Facial expression, Eye-tracking technology, Fixation, ocular, Analog pain scale, Recém-nascido, Medição da dor, Expressão facial, Tecnologia de rastreamento ocular, Fixação ocular, Escala analógica de dor

## Abstract

**Objective:**

To evaluate the focus of pediatricians’ gaze during the heel prick of neonates.

**Methods:**

Prospective study in which pediatricians wearing eye tracker glasses evaluated neonatal pain before/after a heel prtick. Pediatricians scored the pain they perceived in the neonate in a verbal analogue numerical scale (0=no pain; 10=maximum pain). The outcomes measured were number and time of visual fixations in upper face, lower face, and hands, in two 10-second periods, before (pre) and after the puncture (post). These outcomes were compared between the periods, and according to pediatricians’ pain perception: absent/mild (score: 0–5) and moderate/intense (score: 6–10).

**Results:**

24 pediatricians (31 years old, 92% female) evaluated 24 neonates. The median score attributed to neonatal pain during the heel prick was 7.0 (Interquartile range: 5–8). Compared to pre-, in the post-periods, more pediatricians fixed their gaze on the lower face (63 *vs*. 92%; p=0.036) and the number of visual fixations was greater on the lower face (2.0 *vs*. 5.0; p=0.018). There was no difference in the number and time of visual fixations according to the intensity of pain.

**Conclusions:**

At bedside, pediatricians change their focus of attention on the neonatal face after a painful procedure, focusing mainly on the lower part of the face.

## RESUMO

**Objetivo:**

Avaliar o foco do olhar do pediatra durante a punção do calcanhar de neonatos.

**Métodos:**

Estudo prospectivo no qual pediatras, utilizando óculos de rastreamento visual, avaliaram a dor neonatal antes/depois de uma punção de calcanhar. Os pediatras pontuaram a dor de acordo com a sua percepção por meio de uma escala analógica verbal (0=sem dor; 10=dor máxima). Os desfechos analisados foram o número e o tempo das fixações visuais na face superior, face inferior e mãos, em dois períodos de 10 segundos, antes (PRÉ) e depois da punção (PÓS). Os resultados foram comparados entre os períodos e segundo a percepção da dor do pediatra: ausente/leve (escore: 0–5) e moderada/grave (escore: 6–10).

**Resultados:**

Vinte e quatro pediatras (31 anos, 92% sexo feminino) avaliaram 24 neonatos. A mediana do escore atribuído à dor do recém-nascido durante a punção do calcanhar foi 7,0 (intervalo interquartil: 5–8). Comparado ao período PRÉ, no período PÓS, o maior número de pediatras fixou o olhar na face inferior (63 *vs.* 92%; p=0,036) e o número de fixações visuais foi maior na face inferior (2,0 *vs.* 5,0; p=0,018). Não houve diferença no número e no tempo das fixações visuais de acordo com a intensidade da dor.

**Conclusões:**

À beira do leito, os pediatras mudam seu foco de atenção visual na face do recém-nascido após um procedimento doloroso, focando o olhar principalmente na parte inferior da face.

## INTRODUCTION

Pain in the neonatal period has short-term consequences^1,2^ and long-term developmental impact.^3-6^ Pain assessment is critical to avoid under- and overtreatment of neonatal pain. Methods of real-time pain measurement do not exist for neonates and clinicians must rely on non-specific behavioral responses and physiological markers.^7-10^

Understanding the process that drives professionals to report that a neonate is experiencing pain may help to improve pain assessment tools. One way of studying the process of medical decision during neonatal pain assessment is the evaluation of health professionals’ sight. When adults evaluated facial images of newborns at rest or during a painful procedure in static images on a computer screen, pediatricians focused mainly on neonates’ mouth, forehead, and eyes and less on the nasolabial furrow in order to assess pain.^11^ In another analysis, health professionals focused more on the mouth and on the nasolabial furrow when assessing neonatal pain, compared to non-health professionals.^12^ In addition, the attention on the nasolabial furrow was associated with a greater chance of correctly identifying the presence and the absence of pain.^13^ It is not known whether the same facial regions are focused during neonatal pain assessment, in real life, at bedside.

The aim of this study was to verify, at bedside, the focus of pediatricians’ gaze on healthy newborns before and after a heel puncture. The hypothesis of this research is that pediatricians, when observing newborn infants at bedside, focus their gaze differently before and after a heel puncture.

## METHOD

This prospective study enrolled pediatricians that evaluated neonatal pain during a heel prick, while the focus of their gaze was tracked. A convenience sample of pediatricians working in neonatal units was studied, excluding those with visual impairment. In Brazil, both pediatricians/neonatologists and nurses are responsible for pain assessment in the neonatal intensive or special care unit. After the study, pediatricians whose gaze capture was less than 70% of the assessment time were excluded.^14^ The study was approved by the Research Ethics Committee of Escola Paulista de Medicina – Universidade Federal de São Paulo (No. 3.129.321) and a written informed consent was obtained from the pediatricians and from the family of the newborns. The research was carried out in accordance with The Code of Ethics of the World Medical Association (Declaration of Helsinki) for experiments involving humans. The heel puncture was requested for clinical reasons by the assistant physician and performed with non-pharmacologic analgesia.

Visual tracking of pediatricians before and after the heel puncture for blood glucose determination was obtained by Tobii visual tracking glasses, model Pro Glasses 2 (Tobii Technology AB, Danderyd, Sweden). Visual tracking has been used in health sciences to assess human visual behavior, mainly in the diagnosis of diseases such as autism spectrum disorder,^15,16^ Alzheimer disease,^17^ and Parkinson disease,^18^ and in the training of health professionals, as in medical emergencies^19^ and neonatal reanimation^20,21^ Visual tracking glasses have an infrared lighting system located on the inner face of the lens that illuminates the users’ eyes, four cameras, two for each eye, which detect pupil diameter and movement, and an angular scene camera full-HD that captures the users’ visual field. The equipment operates with an image capture capacity of 60 Hz. Infrared light sources, one for each eye, imperceptible to the human eye, illuminate the users’ eyes, generating reflections on the cornea and pupil, which are captured by the two cameras for each eye, over the observation period. Integrated analysis of recorded information from pupillary reflexes and the users’ visual field allows identifying which points of the visual field the user is looking at.^14^

The experiment was performed in a closed room with controlled lighting (425–459 lux) and temperature. The newborns were positioned in a crib in a supine position, with the head at midline and with their face, hands, and feet undressed. The experiment was performed between feedings. All neonates received analgesia with 2 mL of 25% glucose orally two minutes before the procedure. Pediatricians positioned themselves beside the crib, close to the newborn’s face. A screen placed perpendicularly to the crib prevented pediatricians from looking at the lower limbs of the newborns. Therefore, the pediatricians were blind to the exact moment of the heel puncture, and the potential change in the areas of their visual attention would be possibly related to their perception of neonatal pain. The heel prick procedure included: holding one foot, cleaning the heel with 70% alcohol, performing a heel prick, collecting the blood sample, and hemostasis. The procedure was done by a pediatrician or a nurse technician. Pediatricians were instructed orally by the main researcher to assess pain using eyetracker glasses.

Before starting the experiment, the main investigator assessed the newborns’ sleep and wakefulness state.^22^ After 15 seconds of rest, the heel prick was done, and 15 seconds after the puncture the experiment was ended. After the experiment, pediatricians were asked to score their perception of neonatal pain intensity according to a verbal analogue scale, with zero corresponding to the absence of pain, and ten to the most intense pain.

Using the Tobii Pro Lab software (version: 1.162; Tobii Pro AB, Danderyd, Sweden), recordings of the pediatricians’ visual field and pupillary movements were integrated. Before analyzing the eye tracking outcomes, two snapshots were defined, the first taken one second after the start of the experiment (pre-puncture) and the other, one second after the puncture (post-puncture). They were used as a reference for the evaluation of visual tracking outcomes.

In the pre- and post-puncture snapshots of the newborns, three areas of interest for the assessment of visual tracking outcomes were drawn: upper face (forehead, eyes, nose, nasolabial furrow, and malar region); lower face (nasal filter, cheeks, mouth and chin), and hands. These areas were chosen according to the behavioral items included in neonatal pain assessment tools,^23-25^ but the sensitivity of the equipment did not allow the study of individual elements of the face. Data were analyzed in two 10-second windows in the video: pre-puncture (newborn at rest) and post-puncture (starting immediately after the puncture).

The outcomes evaluated were the number of visual fixations,^26^ and the total time of visual fixations in each area of interest in the pre- and post-puncture periods for each newborn assessed by the pediatricians.

Variables were described in median (Md) and interquartile range (IQR) or in number of events and percentage. The percentage of pediatricians who fixed their gaze on the different areas of interest in the pre- *vs*. post-puncture period was compared using the ꭓ^2^ test. The number of fixations and the total time of visual fixations were compared between periods for each area of interest by the Wilcoxon-Rank test for related samples. To analyze the difference in the distribution of visual tracking outcomes, a general linear model for repeated measurements was used considering two factors: period (pre-and post-puncture) and areas of interest (upper face and lower face of the newborn). The number of fixations and the total time of visual fixations in the different areas of interest in the post-puncture period were also compared according to the pain score reported by the pediatrician through Mann-Whitney test.

Sample size calculation was based on data obtained in the study of Silva et al.^11^ Considering a difference in the fixation time in the areas of interest of 0.35 seconds and a standard deviation of 0.70 seconds, with an alpha error of 5%, and a sample power of 80%, it would be necessary to enroll 34 pediatricians in the present research. Estimating a 20% loss, 40 pediatricians should be studied.

All statistical analyses used the SPSS program for Win/v.20.0 (IBM SPSS Statistics, Somers, NY).

## RESULTS

During the study period, 40 pediatricians were invited to participate in the study and 16 of them were excluded: one because the equipment was not calibrated, 11 because the capture of the ocular signal was less than 70%, and four because they did not fix the gaze on the areas of interest. The 24 pediatricians studied were younger than those that were excluded [31.0 (IQR: 29.0–36.0) *vs*. 37.0 (IQR: 32.5–41.0); p=0.036], but similar regarding female gender (91.7 *vs*. 93.3%; p=1.000), years since medical graduation [Md 5.5 (IQR: 4.0–11.3) *vs*. 12.0 (5.0–15.00); p=0.146], years since residency in Pediatrics [Md 2.5 (IQR: 1.0–9.0) *vs*. 8.0 (0.9–11.00); p=0.432], and years working in neonatal intensive care [Md 3.0 (IQR: 1.9–7.3) *vs*. 5.0 (2.5–11.00: p=0.432]. Ten (41.7%) pediatricians concluded residency in Neonatology 7.0 (5.0–10.0) years ago. The workload in the ICU was 55.0 (29.0–60.0) hours and in rooming-in, 41.0 (25.0–60.0) hours. The pediatricians were recruited in two hospitals and in one of them pain scales are routinely used by neonatologists, neonatal fellows, residents, and nurses. Previous personal experience with hospitalized children was reported by 9 (35.5%) pediatricians.

The pediatricians evaluated 24 newborns. They had a mean gestational age of 39.0±1.5 weeks, birth weight of 3,258±666 grams, 2 (8.3%) of them were small and 9 (37.5%) were large for their gestational age; 15 (62.5%) were female. Regarding previous exposure to painful procedures, 24 (100.0%) neonates had received intramuscular vitamin K, 17 (70.8%) intramuscular hepatitis B vaccine, one (4.2%) had undergone a vascular puncture for blood collection, and 23 (95.8%) received heel pricks for blood glucose determination. At the time of the experiment, median postnatal age was 24 hours (IQR 17–32). The patients’ sleep and wakefulness state before starting the procedure were: deep sleep/light sleep/sleepy in 12 of them (50.0%), awake/active in 10 (41.7%), and crying in 2 (8.3%). The pain score reported by pediatricians at the end of the experiment was: 0–5 (absent/mild pain) in 9 (37.5%), and 6–10 (moderate/severe pain) in 15 (62.5%) neonates. Median score was 7 (IQR: 5–8).

The median video review time, in seconds, was similar in the pre-puncture (9.10; IQR 8.59–9.50) *vs*. post-puncture (8.71; IQR 7.96–9.50) periods (Wilcoxon-Rank test; p=0.052). Gaze fixation time, in seconds, on the areas of interest (upper face, lower face, and hands) were also similar in the pre-puncture (8.43; IQR 6.37–9.40) *vs*. post-puncture (7.56; IQR 6.04–9.21) periods (Wilcoxon-Rank test; p=0.230). In the pre-puncture period, pediatricians performed 10.0 fixations (IQR 8.8–16.3) in the areas of interest and, in the post-puncture period, 13.0 fixations (IQR 10.8–18.5) (Wilcoxon-Rank test; p=0.122).

Table 1 shows that a greater number of pediatricians focused their gaze on the lower face in the post-puncture compared to the pre-puncture period. Table 2 shows that pediatricians made a greater number of visual fixations on the lower face in the post-puncture compared to the pre-puncture period.

**Table 1.** Number of pediatricians who fixed their gaze on each area of interest, in the pre-puncture and post-puncture periods.

	Upper face	Lower face	Hands^†^
Pre-Puncture	21/24 (87.5%)	15/24 (62.5%)	5/19 (26.3%)
Post-Puncture	19/24 (79.2%)	22/24 (91.7%)	8/20 (40.0%)
p-value*	0.701	0.036	0.501

* ꭓ^2^ test; ^†^Hands were not visible in five assessments in the pre-puncture period and in four assessments in the post-puncture period.

**Table 2.** Number of fixations and total time of visual fixations in each area of interest, in the pre-puncture and post-puncture periods.

	Upper face	Lower face	Hands
Number of visual fixations			
Pre-puncture period	5.50 (2.80–10.30)	2.00 (0.00–6.00)	0.00 (0.00–1.00)
Post-puncture period	7.00 (1.00–10.30)	5.00 (1.80–10.0)	0.00 (0.00–1.30)
p-value*	0.493	0.018	0.833
Total time of visual fixations (seconds)			
Pre-puncture period	4.30 (1.05–7.55)	1.34 (0.00–4.96)	0.00 (0.00–0.22)
Post-puncture period	3.42 (0.14–5.53)	2.19 (1.28–5.02)	0.00 (0.00–0.23)
p-value*	0.121	0.107	0.859

Data expressed in median (interquartile range). *Wilcoxon-Rank test for related samples

**Figure 1.**
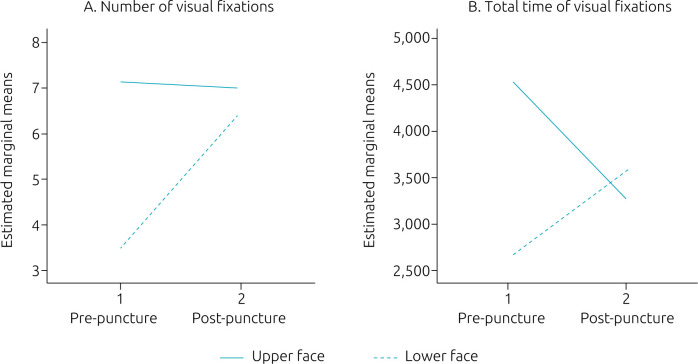
Estimated marginal means of: (A) number of visual fixations, and (B) total time of visual fixations in the upper face (continuous line) and in the lower face (dashed line), in the pre-puncture and post-puncture periods.

Hands were not included in the general linear model, as only 26.3 and 40.0% of pediatricians focused their gaze on this area of interest, respectively in the pre- and post-puncture periods. Although the median number of visual fixations on the lower face increased in the post-puncture period, compared to the upper face (Table 3 and Figure 1), no differences were observed for the main effect period [F(1.23)=3.924; p=0.060] and area of interest [F(1.23)=1.853; p=0.187] or for the interaction between them [F(2.23)=1.574; p=0.222]. When evaluating the estimated marginal means, there was a difference between the number of fixations on the lower face between pre- and post-puncture periods (2.96±1.08; p=0.012) and there was a difference in the number of fixations in the pre-puncture period between the upper face and the lower face (3.67±1.70; p=0.041), with no other differences. Regarding the total time of visual fixations, although the mean visual fixation time increased on the lower face in the post-puncture period, compared to the upper face (Table 3 and Figure 1), no differences were observed for the main effect period [F(1.23)=0.865; p=0.362] and area of interest [F(1.23)=0.625; p=0.437], nor for the interaction between them [F(2.23)=2.038; p=0.167]. No differences were observed in the marginal means in any of the comparisons.

**Table 3.** Linear General Models: Estimated marginal means of the number and total time of fixations in the upper and lower face, in the pre-puncture and post-puncture periods.

	Upper face	Lower face
Number of visual fixations		
Pre-puncture period	7.17±5.72	3.50±4.09
Post-puncture period	7.04±7.40	6.46±5.53
Total time of visual fixations		
Pre-puncture period	4.55±3.49	2.76±3.34
Post-puncture period	3.27±3.02	3.52±3.22

Values expressed as mean ± standard deviation

Finally, there was no difference between the number of visual fixations and the total time of visual fixations by the pediatricians in the areas of interest according to their perception of pain intensity (Table 4).

**Table 4.** Comparison of visual tracking outcomes in areas of interest in the post-puncture period, according to pediatricians’ perception of pain intensity.

	Pain intensity perception		p-value*
	Absent/mild (n=9)	Moderate/intense(n=15)	
Upper face			
Number of visual fixations	1.00 (0.00–10.00)	8.00 (3.00–10.00)	0.423
Total time of visual fixations (seconds)	0.46 (0.00–3.86)	5.18 (0.99–5.56)	0.400
Lower face			
Number of visual fixations	10.00 (1.00–10.00)	5.00 (3.50–7.50)	0.675
Total time of visual fixations (seconds)	3.24 (0.16–9.22)	1.98 (1.50–3.70)	1.000
Hands			
Number of visual fixations	0.00 (0.00–0.50)	0.00 (0.00–3.00)	0.642
Total time of visual fixations (seconds)	0.00 (0.00–0.09)	0.00 (0.00–0.46)	0.642

Data expressed as median and interquartile range. *Mann-Whitney test.

## DISCUSSION

This study evaluated the focus of visual attention of pediatricians when assessing neonatal pain elicited by a heel prick at the bedside. Most pediatricians focused their gaze on the upper and lower face of the infant. In the post-puncture period, compared to pre-puncture, a greater number of pediatricians focused their gaze on the lower face, and the number of visual fixations in this area was also greater. However, this result was not confirmed by the general linear model, probably due to the small sample size. We did not find any differences in the outcomes according to the pediatricians’ perception of neonatal pain intensity.

Previous studies of adults’ gaze during evaluation of pain presence on still images of newborns’ faces showed that, although adults look mainly to newborns’ mouth, eyes, and forehead, the focus of attention on the nasolabial furrow is associated with a greater chance of correctly identifying the presence and absence of pain.^13^ Movements of the neonatal face, such as protrusion of the forehead, narrowing of palpebral fissures, deepening of nasolabial furrows, opening and stretching the mouth, have a sensitivity of 59-89% and a specificity of 87-91% in the identification of neonatal pain.^27^ All these facial movements may help providers to evaluate neonatal pain before, during, and after a skin-breaking procedure; however, the movement of the mouth is the most sensitive indicator of pain.^28^ By evaluating videos of newborns taken before and after painful procedures, Schiavenato et al. noted that the variation in the distance between the extremities of the mouth (horizontal stretch) or between the upper and lower margins of the lips (vertical stretch) was greater than the variation in the distance between the midpoint of the eyebrow and the lower eyelid, indicating that the movement of the mouth in response to a painful procedure has greater amplitude than the movement of the eyes and probably attracts more attention from the observer,^29^ which may justify the greater focus of attention on the lower face in the post-puncture period, noted in our study.

Regarding the upper limbs, Romantsik et al.^25^ showed that facial expressions, hand movements, and duration of crying in full-term newborns are useful for recognizing acute pain in healthy neonates. In preterm infants, flexion of the arms and legs, extension of the legs, opening the fingers and moving the hand toward the face were associated with the pain elicited by a heel prick.^23,24^ In the present study, less than half of the pediatricians focused their attention on the newborns’ hands in order to assess pain. The fact that all studied pediatricians were trained to assess pain with the Neonatal Pain, Agitation and Sedation Scale (N-PASS),^30^ which does not include hand assessment, may have contributed to this finding.

When assessing pain using the verbal analogue scale after the experiment, the scores showed a wide variation that may be associated to factors related to the procedure, to the newborn, to the health professional in charge of pain assessment and to the tool used for this evaluation. Previous experience of pain may modify pain responses, with a cumulative effect of previous procedures in exacerbating the neonatal expression of pain.^1,31^ In the present study, infants had received a median of five painful procedures prior to the experiment, ranging from one to 13, and some variation in the expression of pain may be associated with this variability.

The visual tracking of pediatricians when observing newborn infants after the heel prick was evaluated, according to their perception of pain intensity, classified as absent (score 0–2), mild (score 3–5) and moderate/intense (score ≥6).^32^ There was no association between the visual outcomes and the neonatal pain intensity reported by pediatricians in the post-puncture period. The small sample size was probably an important limitation of this analysis and further studies should evaluate whether there is any difference in the focus of attention of health professionals according to their perception of pain intensity.

This study evaluated the focus of visual attention of pediatricians when assessing neonatal pain elicited by a heel prick at the bedside. Most pediatricians fixed their gaze on the upper and lower face of the infant. In the post-puncture period, compared to pre-puncture, a greater number of pediatricians fixed their gaze on the lower face, and the number of visual fixations in this area was also greater. However, this result was not confirmed by the general linear model. Therefore, this study showed that pediatricians, when assessing newborns’ pain at the bedside during painful procedures, tend to diverge their gauze to the lower part of the face, probably due to the presence of movements of greater amplitude in the mouth area in response to pain, compared to squinting. The diversion of the pediatricians’ visual attention after the puncture to the infants’ lower face can also be due to the movement of the mouth that accompanies crying, although, in response to a painful procedure, the newborn may not cry and open his mouth, or even stretch it vertically or horizontally. These findings confirm the high sensitivity of this area of the face in identifying neonatal pain. However, as the general linear model results suggest, adults in general, and health professionals in special, seem to perceive the presence of pain looking at the whole set of facial expression rather than a very specific facial movement. Adults seems to adopt a holistic viewing strategy to extract expressive cues from all internal facial features in processing naturalistic facial expressions.^33^

Some limitations of this study need to be recognized. The main limitation was the sample size. Among the 40 pediatricians enrolled in the study, 16 were excluded: in one it was not possible to calibrate the equipment, in 11 the capture of the ocular signal occurred in less than 70% of the time of the experiment, and in 4 the pediatricians did not fix their gauze on the areas of interest. Failure to capture the ocular signal can occur due to movement of the glasses users’ head, since it can generate nystagmus-type compensatory eye movements, or during extension or flexion of the trunk to better visualize the newborn, or even if the pediatricians did not focus the gaze through the lenses and adopted a peripheral vision.^34^ Failure to fix the gauze on the areas of interest may be due to a deviation in the focus of the gauze, when the signal of one eye is not captured, or by divergence of gauze between the right and left eyes.^35^ Despite the sample loss, the post-hoc analysis showed a sample power of 70% for the difference between the number of visual fixations on the lower face in the post -puncture period, compared to the pre-puncture period.

Another technical problem was the inability to assess the visual attention in each element of the face. In addition, our study evaluated healthy newborn infants, and it is not possible to generalize the findings for pain evaluation of critically ill neonates. Despite these limitations, our study is innovative, being the first to verify the focus of visual attention of pediatricians using eye tracking glasses during a neonatal pain evaluation *in vivo*, at bedside. Understanding how health professionals perceive the presence and the intensity of pain of pre-verbal patients may be useful to improve the tools to assess neonatal pain, considering face and limbs, and to improve the training of health professionals to apply these tools.^36^ The study of the process that leads health professionals to diagnostic or treatment decisions is an interesting way of understanding how underestimated patients’ complaints may be overcome.

During bedside pain evaluation of healthy newborn infants submitted to a heel prick, more pediatricians focus their visual attention on the face than on the hands. After heel puncture, the number of visual fixations on the lower face was greater, compared to the pre-puncture period, a difference not observed for the upper face and hands. There was no difference in the visual focus of pediatricians according to their perception of neonatal pain intensity.
